# A New Journal

**Published:** 1861-01

**Authors:** 


A NEW JOURNAL.—We have received the first number of the " American Journal of Indigenous Materia Medica," a monthly issue of thirty-two pages, edited by B. Keith, M.D., of New York.

